# Clinical evaluation and validation of laboratory methods for the diagnosis of *Bordetella pertussis* infection: Culture, polymerase chain reaction (PCR) and anti-pertussis toxin IgG serology (IgG-PT)

**DOI:** 10.1371/journal.pone.0195979

**Published:** 2018-04-13

**Authors:** Adria D. Lee, Pamela K. Cassiday, Lucia C. Pawloski, Kathleen M. Tatti, Monte D. Martin, Elizabeth C. Briere, M. Lucia Tondella, Stacey W. Martin, on behalf of the Clinical Validation Study Group

**Affiliations:** 1 IHRC Inc., contracting agency to the Division of Bacterial Diseases, National Center for Immunization and Respiratory Diseases, Centers for Disease Control and Prevention, Atlanta, Georgia, United States of America; 2 Division of Bacterial Diseases, National Center for Immunization and Respiratory Diseases, Centers for Disease Control and Prevention, Atlanta, Georgia, United States of America; Universidad Nacional de la Plata, ARGENTINA

## Abstract

**Introduction:**

The appropriate use of clinically accurate diagnostic tests is essential for the detection of pertussis, a poorly controlled vaccine-preventable disease. The purpose of this study was to estimate the sensitivity and specificity of different diagnostic criteria including culture, multi-target polymerase chain reaction (PCR), anti-pertussis toxin IgG (IgG-PT) serology, and the use of a clinical case definition. An additional objective was to describe the optimal timing of specimen collection for the various tests.

**Methods:**

Clinical specimens were collected from patients with cough illness at seven locations across the United States between 2007 and 2011. Nasopharyngeal and blood specimens were collected from each patient during the enrollment visit. Patients who had been coughing for ≤ 2 weeks were asked to return in 2–4 weeks for collection of a second, convalescent blood specimen. Sensitivity and specificity of each diagnostic test were estimated using three methods—pertussis culture as the “gold standard,” composite reference standard analysis (CRS), and latent class analysis (LCA).

**Results:**

Overall, 868 patients were enrolled and 13.6% were *B*. *pertussis* positive by at least one diagnostic test. In a sample of 545 participants with non-missing data on all four diagnostic criteria, culture was 64.0% sensitive, PCR was 90.6% sensitive, and both were 100% specific by LCA. CRS and LCA methods increased the sensitivity estimates for convalescent serology and the clinical case definition over the culture-based estimates. Culture and PCR were most sensitive when performed during the first two weeks of cough; serology was optimally sensitive after the second week of cough.

**Conclusions:**

Timing of specimen collection in relation to onset of illness should be considered when ordering diagnostic tests for pertussis. Consideration should be given to including IgG-PT serology as a confirmatory test in the Council of State and Territorial Epidemiologists (CSTE) case definition for pertussis.

## Introduction

Pertussis is a highly contagious, prolonged respiratory illness caused by the Gram-negative bacterium *Bordetella pertussis*. Despite high childhood vaccination rates [1, 2], pertussis is one of the most poorly controlled bacterial vaccine-preventable diseases in the United States [3]. The annual incidence of pertussis has increased dramatically in recent years; 48,277 cases were reported in 2012, which is the highest number of cases reported since 1955 [4]. Incidence is highest among infants less than one year of age, who are too young to be fully vaccinated [4]. Additionally, over 90% of pertussis-related deaths occur in this age group [5].

To meet the Council of State and Territorial Epidemiologists (CSTE) clinical case definition, a person must have a cough illness lasting two or more weeks and at least one characteristic pertussis symptom: paroxysmal coughing, inspiratory whoop, or post-tussive vomiting [5]. Diagnosing pertussis is challenging because other respiratory pathogens cause pertussis-like illness, and the clinical presentation of pertussis varies with age and vaccination status [6, 7]. Thus, clinically accurate laboratory diagnostic tests are essential for the confirmation of infection by *B*. *pertussis*.

Due to its 100% specificity, culture is considered the “gold standard” pertussis diagnostic test. However, it has very low sensitivity, with estimates ranging from 12%-60% [8], and results may take 1–2 weeks to obtain [9]. To obtain viable bacteria for isolation, nasopharyngeal (NP) specimens should be collected within the first two weeks of illness, when symptoms are more likely to be non-specific and physicians might not consider pertussis as the diagnosis. Additionally, the organism can be difficult to isolate, especially if the patient has been recently vaccinated or received antibiotics against pertussis [10].

Polymerase chain reaction (PCR) is the most commonly used pertussis diagnostic test in the United States [11]. Real-time PCR (RT-PCR) can be completed in two to 24 hours, which allows for rapid diagnosis. Since PCR does not require the collection of viable bacteria to be positive, it is more sensitive than culture, with estimates ranging from 70%-99% when performed during the first three to four weeks of cough [12, 13]. Multi-target RT-PCR assays are used to differentiate between *Bordetella* species, and can be highly specific [14]. However, environmental contamination of clinical specimens in clinics and cross-contamination within laboratories has been associated with false positive PCR results and several pseudo-outbreaks of pertussis in recent years [15–17].

Although not included in the CSTE case definition, serologic tests are increasingly used to diagnose recent pertussis infections. Currently, the lack of commercially available, validated and standardized serodiagnostic assays limits their use in routine clinical practice in the United States and elsewhere [18, 19].

The Centers for Disease Control and Prevention (CDC) Pertussis and Diphtheria Laboratory has developed its own PCR and serological assays to diagnose pertussis. The multi-target RT-PCR assay combines a pertussis toxin subunit 1 (*ptxS1*) singleplex assay and a multiplex assay targeting the insertion sequence *481* (IS*481*), *B*. *parapertussis* IS*1001* (pIS*1001*), and *B*. *holmesii* IS*1001*-like (hIS*1001*) to distinguish between *Bordetella* species and identify co-infections. Specimens with IS*481* cycle threshold (Ct) values in the range 35–40 are classified as indeterminate for *B*. *pertussis* infection, as this range indicates specimens containing less than one bacterium per reaction, which may either be truly positive, or falsely positive due to contamination [14]. The anti-pertussis toxin IgG (IgG-PT) serological enzyme-linked immunosorbent assay (ELISA) is specific for *B*. *pertussis* and has been used to confirm pertussis as the causative agent of recent outbreaks [15, 20, 21]. It has been analytically validated using the World Health Organization (WHO) pertussis reference standard, and a study in adults suggests it can be used to diagnose pertussis as early as six months following receipt of Tdap, the adolescent and adult pertussis toxin-containing booster vaccine, as vaccine-induced antibody titers are expected to fall below the diagnostic cutoff by this time [18, 22].

Imperfect pertussis diagnostics may contribute to an underestimation or overestimation of disease burden across the age spectrum, and compromise prevention programs, surveillance activities, vaccine effectiveness studies, and outbreak management. For these reasons, we assessed the relative clinical validity by time since cough onset of CDC’s array of pertussis diagnostics by evaluating the sensitivities and specificities of culture, multi-target RT-PCR, and IgG-PT serology for the diagnosis of *B*. *pertussis* infection.

## Methods

### Study population

Individuals at least three years of age who met the inclusion criteria listed in Table 1 were invited to participate in the study. Participants were recruited from July 2007 to February 2011 by state and local public health investigators through routine surveillance and outbreak investigations, and by Emerging Infections Programs (EIP) investigators collaborating with health management organizations and private and public healthcare providers. Participants were identified through prospective reviews of hospital admissions, laboratory test orders, emergency department visits, and/or outbreak investigations in California, Colorado, Georgia, Minnesota, New Mexico, and New York. Participants were also recruited by CDC employees during outbreak investigations beginning in 2008.

**Table 1:** Enrollment criteria for participation in the clinical validation study, 2007–2011.

1	Cough 5–29 days duration, or
2	Cough < 5 days duration with at least one of the following “classical” pertussis symptoms: Paroxysms of coughing Inspiratory “whoop” Post-tussive vomiting, or
3	Close contact^a^ of a Council of State and Territorial Epidemiologists (CSTE)- or PCR-confirmed case, plus cough < 30 days duration

^a^Close contacts are persons who have shared a confined space of ≤ 3 feet for at least 1 cumulative hour per day with a confirmed case or have direct contact with respiratory secretions from a confirmed case.

Demographic and clinical information, including vaccination history, presence of pertussis symptoms, duration of cough illness, and recent use of antibiotics, were collected from each participant during the enrollment visit.

### Ethics statement

The study was approved by the Centers for Disease Control and Prevention Institutional Review Board (IRB; #5029), Children’s Hospital and Research Center Oakland IRB (#2007–054), Committee for the Protection of Human Subjects, California Health and Human Services Agency (#07-04-03), Kaiser Permanente Northern California IRB (#CN-07TGree-02-H), Kaiser Permanente of Colorado IRB (#CO-07MRaeb-01), Georgia Department of Human Resources IRB (#070208), Kaiser Permanente Georgia IRB (#GA-07RDavi-01), Minnesota Department of Health IRB (#07–159), The University of New Mexico Health Sciences Human Research Review Committee (#07–273), and the Biomedical Research Alliance of New York IRB, Montefiore Medical Center (#07-10-245-01). Each enrolled participant provided written informed consent. Written parental permission was obtained for participants less than 18 years of age in addition to adolescent assent for participants aged 11–18 years.

### Specimen collection and preparation

A NP aspirate or swab specimen and a blood specimen were collected from each participant during the enrollment visit. Swabs were immediately stored in tubes containing 2 mL of 1% casamino acid broth and vortexed for five minutes in a biosafety cabinet (BSC). Within 24 hours of specimen collection, the NP aspirate or swab suspension was divided into 300 μL aliquots in a *Bordetella* species DNA-free BSC in a room where no culture was performed. One aliquot was reserved for culture by the site laboratory, and the others were stored at −40°C to −80°C until shipped to CDC on dry ice for PCR testing. For NP aspirates collected by CDC employees during outbreak responses, all aliquots were shipped overnight to CDC for culture and PCR. Whole blood was allowed to clot for 30–45 minutes at room temperature and was centrifuged within two hours of collection. The serum was separated and divided into 300 μL aliquots within 24 hours of blood collection. Aliquots were stored at −40°C to −80°C until shipped to CDC on dry ice. Participants who had been coughing for ≤ 2 weeks at enrollment (i.e., 1–14 days) were asked to return in two to four weeks for collection of a second, convalescent blood specimen which was processed similarly.

### Laboratory diagnostic tests

NP specimens were plated onto Regan-Lowe agar plates with and without cephalexin, which were incubated at 37°C with high humidity under ambient air and examined daily for seven to ten days. Colonies were stained to check for Gram-negative coccobacilli, and *Bordetella* species were confirmed by direct fluorescent antibody (DFA) testing, slide agglutination using specific antisera, and biochemical tests, as available, at each laboratory site. dx.doi.org/10.17504/protocols.io.kq9cvz6; dx.doi.org/10.17504/protocols.io.kvtcw6n.

Multi-target RT-PCR was performed at CDC as previously described [12, 14]. dx.doi.org/10.17504/protocols.io.kvxcw7n.

IgG-PT ELISAs were performed at CDC as previously described [18]. Blood specimens collected ≤ 2 weeks after cough onset were classified as “acute” specimens, and specimens collected > 2 weeks after onset were classified as “convalescent” specimens. For all specimens, antibody concentrations ≥ 94 EU/mL were considered positive for pertussis, concentrations 49–93 EU/mL were considered indeterminate, and concentrations < 49 EU/mL were considered negative [23]. Log-transformed antibody concentrations were calculated as log_10_ (antibody concentration +1). dx.doi.org/10.17504/protocols.io.kvwcw7e.

### Sensitivity and specificity analyses

Statistical analyses were conducted using SAS 9.3 (SAS Institute, Inc., Cary, NC) and Latent Gold 4.0 (Statistical Innovations, Inc., Belmont, MA). Sensitivities and specificities of PCR, acute and convalescent serology, and the clinical case definition were estimated using culture as the “gold standard” test. However, due to its low sensitivity, culture is an imperfect gold standard and this method may produce biased estimates [24]. Therefore, sensitivities and specificities were also estimated using composite reference standard (CRS) analysis and latent class analysis (LCA).

CRS reduces bias by combining the results of the imperfect gold standard and a more sensitive diagnostic measure to create a new reference standard [24]. The CRS was created by combining culture and PCR results and was used to estimate the sensitivity and specificity of acute and convalescent serology and the clinical case definition. If either culture or PCR was positive, the CRS was defined as positive. If both tests were negative, the CRS was defined as negative. If one test result was negative and the other was missing, the CRS was defined as missing.

LCA was used to estimate the sensitivity and specificity of each diagnostic measure and the prevalence of pertussis in the study population. In LCA, gold standard bias is reduced by considering all diagnostic measures as imperfect. The statistical model combines the results of each measure to define an unmeasured latent variable that indicates true disease status. The model calculates the probability of each participant being classified as a case or a non-case, as well as the overall probability of participants being classified as cases (i.e., the prevalence of pertussis within the study population), based on participants’ results on at least three diagnostic measures and an assumption of conditional independence between the measures. A bivariate residual (BVR) substantially greater than 1 indicates a violation of this assumption [25]. To address this issue, a conditional dependence model is fit that includes a direct effect between two statistically associated measures. Good model fit is indicated by low, non-significant likelihood ratio statistics (L^2^) and a low proportion of classification errors [25].

Sensitivities and specificities were estimated among participants with non-missing data on culture, PCR, serology, and the clinical case definition. Several models were included to assess the effect of the timing of specimen collection on the sensitivity and specificity estimates. Model 1 included participants with all specimens collected 1–29 days after cough onset, Model 2 included participants with all specimens collected > 2 weeks after cough onset, and Model 3 included participants with all specimens collected ≤ 2 weeks after onset. Model 4 included participants with NP specimens collected ≤ 2 weeks after onset and a blood specimen collected 2–4 weeks later. Models 5A and 5B included participants with NP and blood specimens collected ≤ 2 weeks after onset, and a second blood specimen collected 2–4 weeks later.

## Results

### Demographics and clinical characteristics

A total of 868 persons with cough illness were enrolled in the study between July 2007 and February 2011 (Table 2). The mean age at enrollment was 32 years (range: 3–83 years), and participants enrolled in the study an average of 14 days after their cough onset (range: 1–29 days). Overall, 58.5% (508/868) of participants experienced at least one characteristic pertussis symptom. Of the 310 (35.7%) participants who responded as having been previously vaccinated against pertussis, 96.1% (298/310) were able to provide the date of their most recent vaccination. The median time between their most recent receipt of a pertussis-containing vaccine and study enrollment was four years (range: 0–40 years). Twenty participants had been vaccinated less than six months prior to their cough onset. Among the 190 participants who reported taking antibiotics prior to their enrollment in the study, the mean time between the start of antibiotic use and study enrollment was 10 days (range: 1–28 days). In addition, participants who reported antibiotic use were more likely to enroll later than those who did not report antibiotic use, on average (16 days vs. 13 days, p-value <0.0001).

**Table 2:** Demographic, clinical, and epidemiological characteristics of all participants enrolled in the clinical validation study (N = 868).

	N (%)
**Demographics**	
Type of study site	
Routine pertussis investigations	731 (84.2)
CDC-initiated outbreak investigations	137 (15.8)
Enrollment year	
2007	41 (4.7)
2008	377 (43.4)
2009	284 (32.7)
2010	145 (16.7)
2011	21 (2.4)
Age at enrollment	
3–10 years	155 (17.9)
11–19 years	110 (12.7)
20–64 years	579 (66.7)
≥ 65 years	24 (2.8)
Sex	
Female	585 (67.4)
Male	281 (32.4)
Missing	2 (0.2)
Race	
White	544 (62.7)
Non-white	278 (32.0)
Missing	46 (5.3)
Ethnicity	
Non-Hispanic	619 (71.3)
Hispanic	174 (20.0)
Missing	75 (8.6)
**Clinical symptoms, vaccination history, and antibiotic use**	
Cough duration at enrollment	
≤ 2 weeks	487 (56.1)
> 2 weeks	381 (43.9)
Paroxysmal coughing	
No	357 (41.1)
Yes	479 (55.2)
Missing	32 (3.7)
Post-tussive vomiting	
No	701 (80.8)
Yes	139 (16.0)
Missing	28 (3.2)
Inspiratory whoop	
No	683 (78.7)
Yes	126 (14.5)
Missing	59 (6.8)
Apnea	
No	701 (80.8)
Yes	118 (13.6)
Missing	49 (5.6)
Meets CSTE clinical case definition	
No	468 (53.9)
Yes	349 (40.2)
Missing	51 (5.9)
Ever received a pertussis-containing vaccine	
No	24 (2.8)
Yes	310 (35.7)
Missing	534 (61.5)
Antibiotic use 1 month before enrollment	
No	647 (74.5)
Yes	190 (21.9)
Missing	31 (3.6)
**Epidemiology**	
Illness related to a pertussis outbreak	
No	483 (55.7)
Yes	305 (35.1)
Missing	80 (9.2)
Contact with a lab-confirmed pertussis case	
No	492 (56.7)
Yes	207 (23.8)
Missing	169 (19.5)

### Laboratory diagnostic test results

The laboratory diagnostic test results are described in Table 3. In total, 13.6% (118/868) of participants were positive for *B*. *pertussis* infection by at least one laboratory diagnostic test. *B*. *pertussis* was isolated from 2.5% of NP specimens, and there were no differences in the proportion of culture-positive specimens among those with and without prior antibiotic use (2.3% vs. 2.9%, p-value = 0.74). Seventeen of the 22 culture-positive participants were also PCR-positive for *B*. *pertussis*. Acute and convalescent blood specimens were collected an average of eight days (range: 1–14 days) and 24 days (range: 15–69 days) after cough onset, respectfully. Of those with positive convalescent serology, 77.4% (65/84) also met the clinical case definition, and 69.0% (58/84) were enrolled > 2 weeks after their cough onset and had negative results by both culture and PCR. Additionally, two convalescent serology-positive participants were vaccinated less than six months prior to cough onset (vaccinated 2.2 months and 5.7 months prior); both reported cough lasting three weeks and were culture- and PCR-negative, and one met the clinical case definition.

**Table 3:** Laboratory diagnostic test results for all participants enrolled in the clinical validation study (N = 868).

	Test Results, N (%)
**Culture**	
Negative	802 (92.4)
*B*. *pertussis*	22 (2.5)
Missing^a^	44 (5.1)
**PCR**	
Negative	758 (87.3)
*B*. *pertussis*	31 (3.6)
Indeterminate *B*. *pertussis*	13 (1.5)
*B*. *parapertussis*	4 (0.5)
*B*. *holmesii*	3 (0.3)
Missing^a^	59 (6.8)
**Acute serology**^b^	
Negative	395 (45.5)
Enrolled >2 weeks after cough onset (missed acute illness)	381 (43.9)
Indeterminate	31 (3.6)
Positive	23 (2.6)
Missing^a^	38 (4.4)
**Convalescent serology**^c^	
Negative	522 (60.1)
Positive	84 (9.7)
Indeterminate	46 (5.3)
Missing^a^	216 (24.9)

^a^ Test results were classified as missing if the participant was eligible for specimen collection, but no diagnostic test result was available.

^b^Acute sera are collected ≤ 2 weeks after cough onset.

^c^ Convalescent sera are collected > 2 weeks after cough onset.

**Fig 1:**
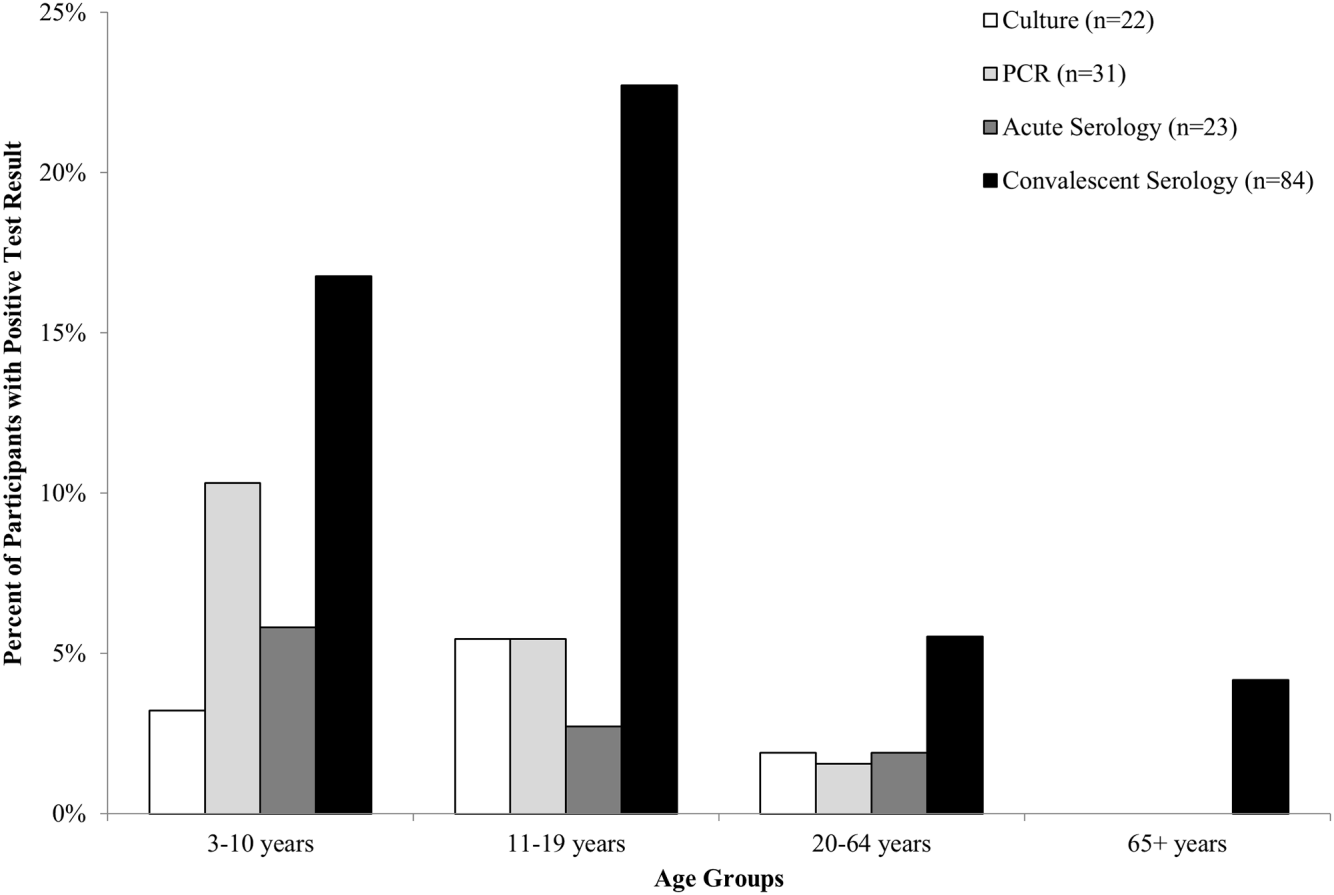
*B*. *pertussis*-positive laboratory test results by age group (N = 868). Acute sera are collected ≤ 2 weeks after cough onset, and convalescent sera are collected > 2 weeks after cough onset.

**Fig 2:**
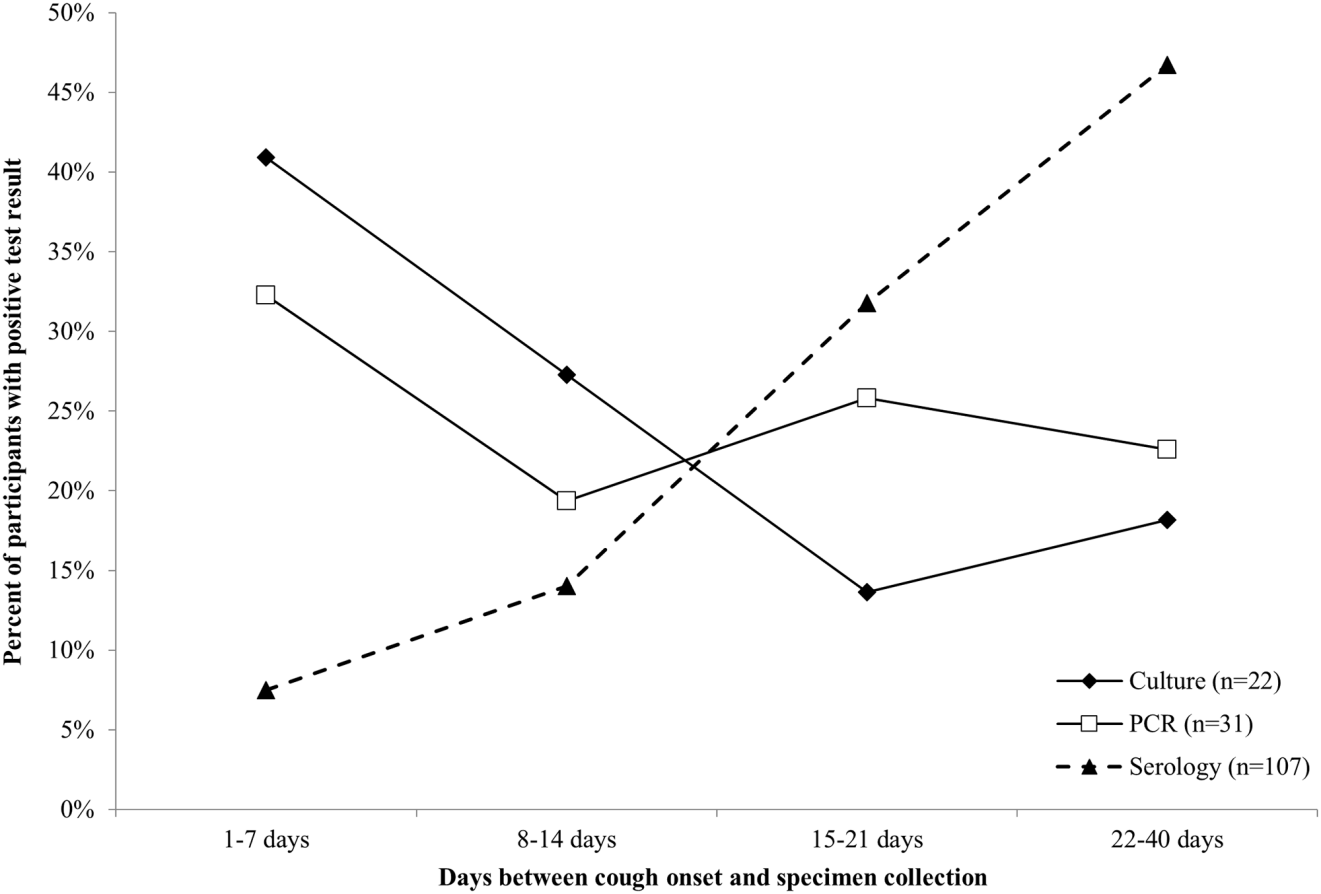
*B*. *pertussis*-positive laboratory test results by time of specimen collection.

Fig 1 shows the proportion of participants in each age group who tested positive for *B*. *pertussis* by culture, PCR, acute serology, and/or convalescent serology. Overall, 61.9% (73/118) of those positive by at least one laboratory test were ≤ 19 years of age. No participants in the 65+ year age group tested positive for *B*. *pertussis* by culture, PCR, or acute serology; one participant was positive by convalescent serology. The effect of the timing of the clinical specimen collection on the laboratory diagnostic test result was also assessed (Fig 2). Overall, 68.2% (15/22) of the culture-positive NP specimens and 51.6% (16/31) of the PCR-positive NP specimens were collected ≤ 14 days after cough onset. In contrast, 78.5% (84/107) of the serology-positive blood specimens were collected 15–40 days after cough onset.

**Fig 3:**
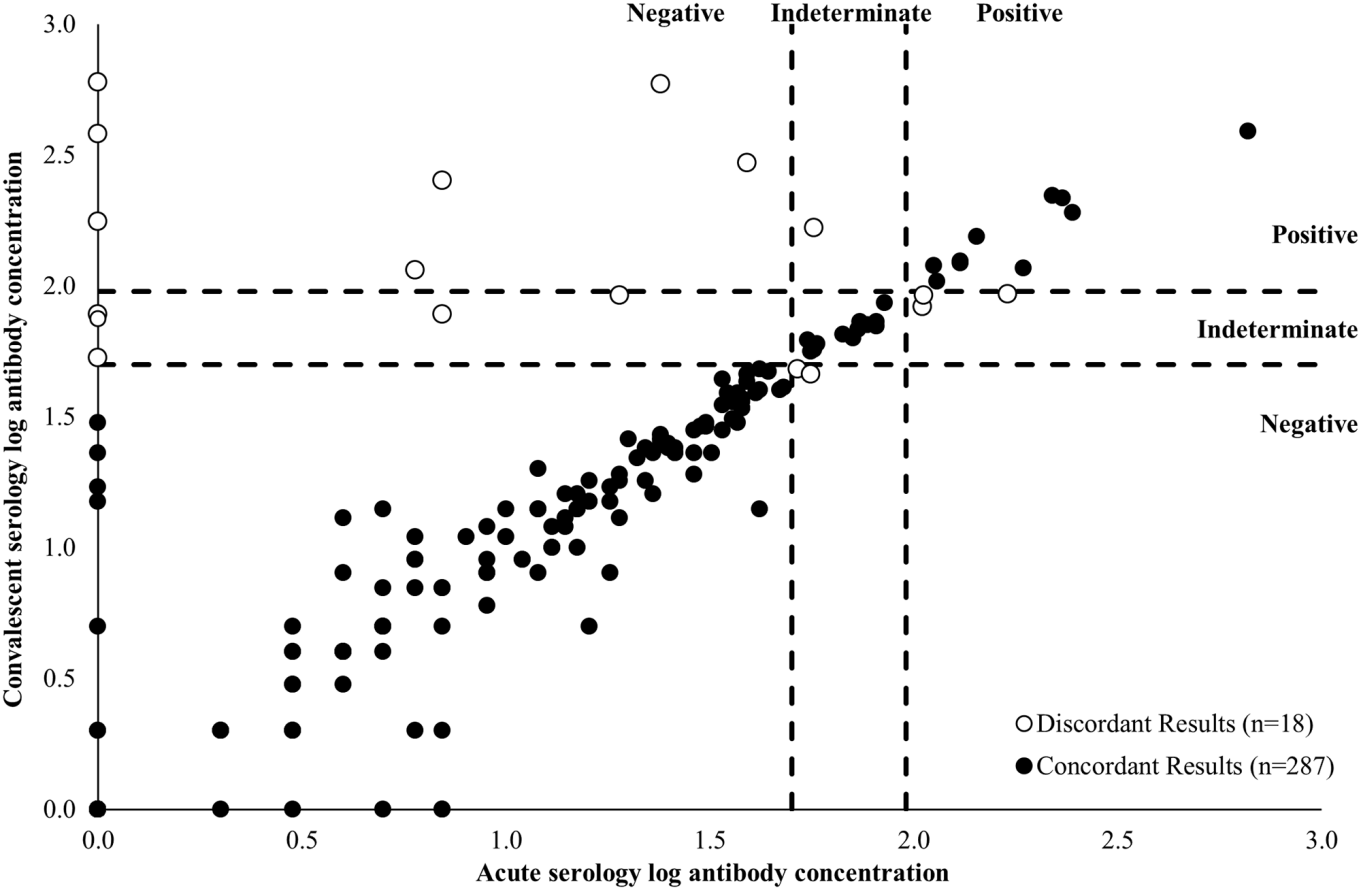
Comparison of log-transformed acute and convalescent serology results (n = 305). Acute sera are collected ≤ 2 weeks after cough onset, and convalescent sera are collected > 2 weeks after cough onset. Open circles indicate participants with discordant results, and shaded circles indicate participants with concordant results at the acute and convalescent time periods. Dashed lines indicate the log-transformed diagnostic cutoff values. Log-transformed values ≥ 1.98 EU/mL were considered positive for pertussis, values 1.70–1.97 EU/mL were considered indeterminate, and values <1.96 EU/mL were considered negative.

**Fig 4:**
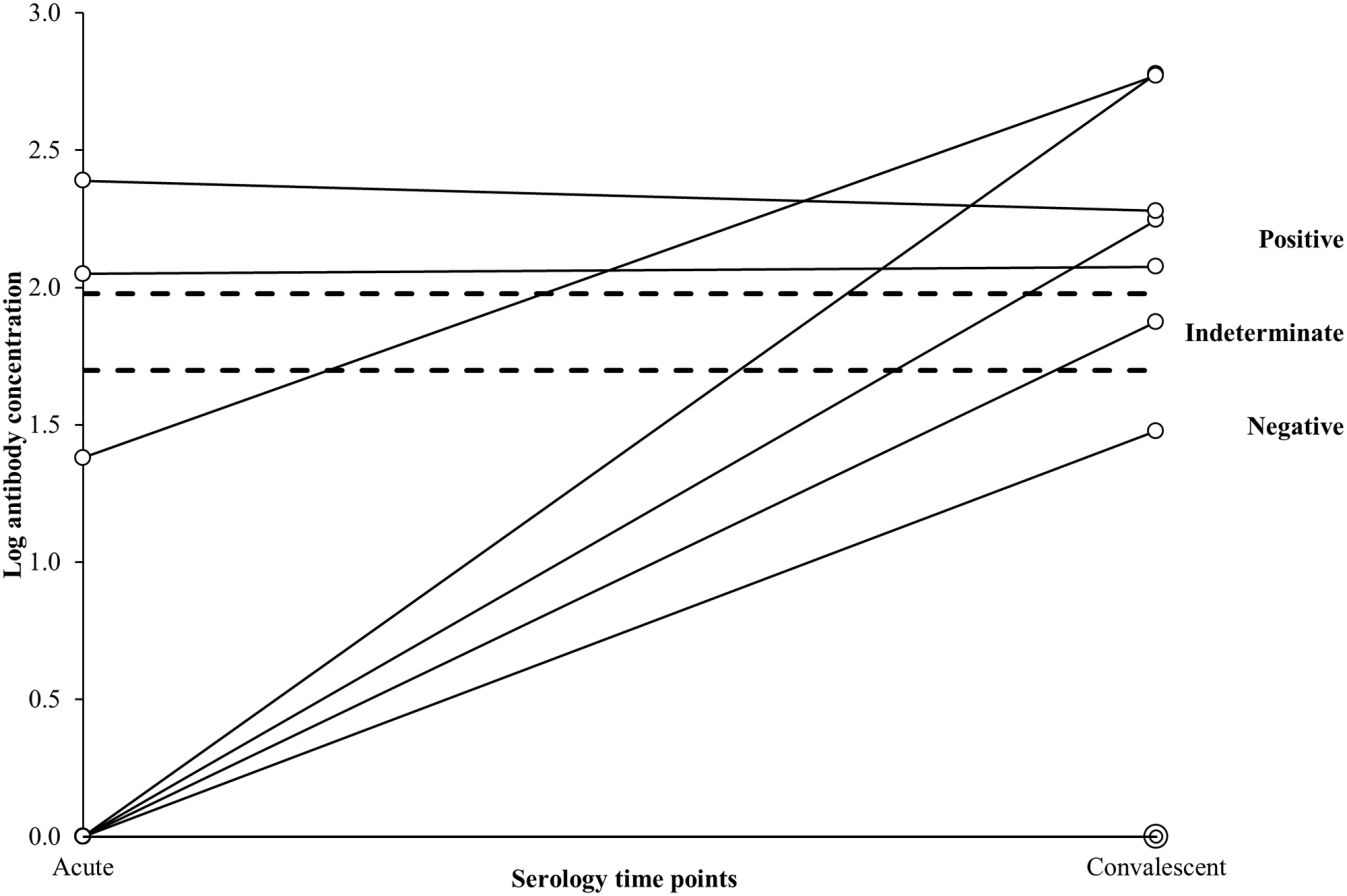
Log-transformed acute and convalescent antibody concentrations of nine *B*. *pertussis* culture-positive participants. Acute sera are collected ≤ 2 weeks after cough onset, and convalescent sera are collected > 2 weeks after cough onset. Two participants had undetectable antibody concentrations at both the acute and convalescent time points. Dashed lines indicate the log-transformed diagnostic cutoff values. Log-transformed values ≥ 1.98 EU/mL were considered positive for pertussis, values 1.70–1.97 EU/mL were considered indeterminate, and values <1.96 EU/mL were considered negative.

Three hundred and five participants provided both an acute and convalescent blood specimen. The median number of days between blood specimen collections was 20 days (range: 13–62 days). The log-antibody concentrations at both time points are shown in Fig 3. Eight of the 18 participants with discordant results between the acute and convalescent draws were only positive at the convalescent time point. Fig 4 shows the log-antibody concentrations of nine participants who were culture-positive and provided both an acute and convalescent blood specimen. On average, their blood specimens were collected 20 days apart (range: 14–26 days). Two participants were positive by serology at the acute time point. By the convalescent time point, five participants were positive and one was indeterminate. Despite being culture-positive, three participants were serology-negative at both time points, including two with undetectable antibody concentrations at both time points (Fig 4). One of these two participants was also PCR-negative for all *Bordetella* species, while the eight remaining participants were PCR-positive for *B*. *pertussis*.

### Sensitivity and specificity analyses

LCA requires participants to have a result for all diagnostic measures included in the model. Therefore, 275 participants with a missing result for at least one diagnostic measure were excluded from the sensitivity and specificity analyses. Additionally, 48 participants with an indeterminate PCR or serology result were excluded to reduce potential misclassification. Two participants who were PCR-positive for *B*. *parapertussis* and two who were PCR-positive for *B*. *holmesii* were categorized as “*B*. *pertussis* PCR-negative.” Sensitivity and specificity estimates are listed in Table 4.

**Table 4:** Sensitivity and specificity estimates of *B*. *pertussis* culture, PCR, serology, and the clinical case definition.

NP specimen collection timeframes	Diagnostic measures	# Positive	Culture as the “gold standard”		Composite reference standard		Latent class analysis	
			Sensitivity (95% CI)	Specificity (95% CI)	Sensitivity (95% CI)	Specificity (95% CI)	Sensitivity (95% CI)	Specificity (95% CI)
**Model 1** 1–29 days after cough onset, n = 545	**Culture**	13	N/A	N/A	N/A	N/A	64.0 (41.8–86.2)	99.9 (99.4–100)
	**PCR**	18	92.3 (77.8–100)	98.9 (98.0–100)	N/A	N/A	90.6 (65.6–100)	100 (99.4–100)
	**Convalescent serology**^a^	72	61.5 (35.1–88.0)	88.0 (85.2–90.7)	73.7 (53.9–93.5)	89.0 (86.3–91.7)	73.2 (50.9–95.6)	89.1 (86.4–91.8)
	**Clinical case**	230	84.6 (65.0–100)	58.8 (54.7–63.0)	89.5 (75.7–100)	59.5 (55.3–63.7)	88.1 (73.5–100)	59.5 (55.3–63.7)
**Model 2** > 2 weeks after cough onset, n = 281	**Culture**	5	N/A	N/A	N/A	N/A	9.7 (0.8–18.7)	100 (99.9–100)
	**PCR**	11	100 (100–100)	97.8 (96.1–99.6)	N/A	N/A	21.4 (7.3–35.5)	100 (99.9–100)
	**Convalescent serology**^a^	57	60.0 (17.1–100)	80.4 (75.8–85.1)	81.8 (59.0–100)	82.2 (77.7–86.8)	78.4 (54.1–100)	92.7 (87.3–98.1)
	**Clinical case**	166	100 (100–100)	41.7 (35.9–47.5)	100 (100–100)	42.6 (36.7–48.5)	98.1 (90.1–100)	49.6 (42.1–57.2)
**Model 3** ≤ 2 weeks after cough onset, n = 347	**Culture**	12	N/A	N/A	N/A	N/A	73.9 (48.7–99.0)	99.9 (99.1–100)
	**PCR**	13	83.3 (62.3–100)	99.1 (98.1–100)	N/A	N/A	81.2 (52.7–100)	100 (99.5–100)
	**Acute serology**^b^	15	16.7 (0.0–37.8)	96.1 (94.1–98.2)	13.3 (0.0–30.5)	96.1 (94.0–98.2)	13.3 (0.0–30.5)	96.1 (94.0–98.2)
	**Clinical case**	83	58.3 (30.4–86.2)	77.3 (72.8–81.8)	60.0 (35.2–84.8)	77.7 (73.2–82.2)	59.7 (33.7–85.6)	77.8 (73.3–82.3)
**Model 4** ≤ 2 weeks after cough onset, n = 264	**Culture**	8	N/A	N/A	N/A	N/A	93.2 (74.6–100)	100 (99.8–100)
	**PCR**	7	87.5 (64.6–100)	100 (100–100)	N/A	N/A	81.7 (54.7–100)	100 (99.9–100)
	**Convalescent serology**^a^	15	62.5 (29.0–96.1)	96.1 (93.7–98.5)	62.5 (29.0–96.1)	96.1 (93.7–98.5)	59.2 (26.1–92.4)	96.1 (93.7–98.5)
	**Clinical case**	64	75.0 (45.0–100)	77.3 (72.2–82.5)	75.0 (45.0–100)	77.3 (72.2–82.5)	71.9 (41.7–100)	77.4 (72.2–82.5)
**Model 5A** ≤ 2 weeks after cough onset, n = 258	**Culture**	8	N/A	N/A	N/A	N/A	93.4 (75.4–100)	100 (99.8–100)
	**PCR**	7	87.5 (64.6–100)	100 (100–100)	N/A	N/A	81.9 (55.2–100)	100 (99.9–100)
	**Acute serology**^b^	10	25.0 (0.0–55.0)	96.8 (94.6–99.0)	25.0 (0.0–55.0)	96.8 (94.6–99.0)	23.9 (0.0–52.6)	96.8 (94.6–99.0)
	**Convalescent serology**^a^	15	62.5 (29.0–96.1)	96.0 (93.6–98.4)	62.5 (29.0–96.1)	96.0 (93.6–98.4)	59.2 (26.0–92.3)	96.0 (93.6–98.4)
	**Clinical case**	62	75.0 (45.0–100)	77.6 (72.4–82.8)	75.0 (45.0–100)	77.6 (72.4–82.8)	71.8 (41.5–100)	77.6 (72.4–82.8)

Abbreviations: NP, nasopharyngeal; 95% CI, 95% Confidence Interval; N/A, not applicable

^a^ Convalescent sera are collected > 2 weeks after cough onset. Participants with NP specimens collected ≤ 2 weeks after cough onset returned 2–4 weeks later to provide the convalescent blood specimen

^b^ Acute sera are collected ≤ 2 weeks after cough onset

#### Model 1

Model 1 includes 545 participants with complete data on pertussis culture, PCR, convalescent serology, and the clinical case definition. Clinical specimens were collected 1–29 days after cough onset. When compared to culture, PCR was the most sensitive and specific diagnostic measure (Table 4). Convalescent serology was the least sensitive, and the clinical case definition was the least specific measure. Use of the CRS method increased the sensitivity estimates for convalescent serology and the clinical case definition (Table 4). Bivariate residuals of the initial LCA model indicated that there was an association between convalescent serology and the clinical case definition (BVR = 19.8). Therefore, a second LCA model was fit that included a direct effect between these two measures, which improved model fit (L^2^ = 8.4; p-value = 0.14). The rate of expected classification errors was 0.2%. PCR was the most sensitive and culture was the least sensitive diagnostic measure in the LCA model (Table 4). The sensitivity and specificity estimates for PCR were similar to those obtained when culture was considered the “gold standard,” and the sensitivity and specificity of both convalescent serology and the clinical case definition were similar to the estimates from the CRS method. The estimated prevalence of pertussis was 3.6% (95% CI: 1.8%- 5.4%). Nineteen participants had a probability of having pertussis ≥ 0.50, and were thus classified as cases (Table 5). The LCA model classified 80.6% (58/72) of participants with positive convalescent serology as non-cases.

**Table 5:** Diagnostic measure result patterns and case classification of participants in Model 1 (n = 545).

Culture	PCR	Convalescent serology^a^	Clinical case	N	Probability of having pertussis	Classification
−	−	−	−	296	0.0001	Non-case (n = 526)
−	−	−	+	172	0.0008	
−	−	+	−	17	0.0021	
−	−	+	+	41	0.0111	
−	+	+	+	6	0.9997	Case (n = 19)
+	−	−	+	1	0.7033	
+	+	−	−	1	1.0000	
+	+	−	+	3	1.0000	
+	+	+	−	1	1.0000	
+	+	+	+	7	1.0000	

Participants in Model 1 enrolled in the study 1–29 days after cough onset. Positive test results are indicated by (+), and negative test results are indicated by (−). Participants with missing data or indeterminate PCR or convalescent serology results were excluded from the analysis. The LCA model contains a direct effect between convalescent serology and the clinical case definition.

^a^Convalescent sera are collected > 2 weeks after cough onset

#### Model 2

Model 2 includes 281 participants with all clinical specimens collected > 2 weeks after cough onset. Only five participants had positive culture results, and all five were PCR-positive and met the clinical case definition (Table 4). The CRS-based sensitivity estimate for convalescent serology was higher than the culture-based estimate, while the specificity estimates for convalescent serology and the clinical case definition were similar to the corresponding culture-based estimates (Table 4). Bivariate residuals indicated an association between culture and PCR in the LCA model (BVR = 15.3), therefore a direct effect between these two measures was included. This model had good fit (L^2^ = 4.3; p-value = 0.51); however, the probability of classification errors was 6.3%, indicating poor separation between the latent classes. Convalescent serology was more sensitive than both culture and PCR in the LCA model (Table 4). The estimated prevalence of pertussis was 18.2% (95% CI: 9.7%- 26.8%). Fifty participants were classified as cases, and 15.8% (9/57) of participants with positive convalescent serology results were classified as non-cases (S1 Table).

#### Model 3

To further assess the effect of the timing of blood specimen collection, 347 participants who had all clinical specimens collected ≤ 2 weeks after cough onset were included in Model 3. Acute serology was the least sensitive diagnostic measure by all three methods (Table 4). The CRS-based sensitivity and specificity estimates for acute serology and the clinical case definition were similar to the culture-based estimates (Table 4). The LCA model had good fit (L^2^ = 4.8; p-value = 0.57), and the probability of classification errors was 0.3%. The estimated prevalence of pertussis was 4.6% (95% CI: 2.02%- 7.12%). Fifteen participants were classified as cases, and 86.7% (13/15) of participants with positive acute serology results were classified as non-cases (S2 Table).

#### Model 4

Of those who enrolled ≤ 2 weeks after cough onset, 264 participants returned for collection of a convalescent blood specimen, and were included in Model 4. The culture- and CRS-based sensitivity and specificity estimates for convalescent serology and the clinical case definition were similar (Table 4). The LCA model had good fit (L^2^ = 4.8; p-value = 0.57), and the probability of classification errors was 0%. Culture was the most sensitive diagnostic measure (Table 4). The sensitivities and specificities of PCR, convalescent serology, and the clinical case definition were similar to the culture- and CRS-based estimates. The estimated prevalence of pertussis was 3.2% (95% CI: 1.1%- 5.4%). Eight participants were classified as cases, and 66.7% (10/15) of participants with positive convalescent serology were classified as non-cases (S3 Table).

#### Models 5A and 5B

To directly compare the sensitivity and specificity of acute and convalescent serology, Models 5A and 5B included 258 participants from Model 4 who had NP and blood specimens collected ≤ 2 weeks after cough onset, and returned for collection of a convalescent blood specimen. There were no differences in the culture- and CRS-based sensitivity and specificity estimates between the two models, and the culture-based estimates for acute and convalescent serology and the clinical case definition were similar to the corresponding CRS-based estimates (Table 4). Bivariate residuals indicated associations between several pairs of diagnostic measures in the LCA model. Thus, two LCA models were run, each controlling for a different pair of associations. LCA Model 5A (Table 4) accounted for the correlation between the serology results by including a direct effect between acute and convalescent serology (BVR = 3.3). This model had good fit (L^2^ = 7.0; p-value = 0.99), and the probability of classification errors was 0%. The LCA-based sensitivity and specificity estimates for PCR, acute serology, convalescent serology, and the clinical case definition were similar to the culture- and CRS-based estimates of Models 5A and 5B and the LCA-based estimates of Model 4 (Table 4). The estimated prevalence of pertussis was 3.3% (95% CI: 1.1%- 5.5%). Eight participants were classified as cases, and 80.0% (8/10) of participants with positive acute serology and 66.7% (10/15) with positive convalescent serology were classified as non-cases (S4 Table).

LCA Model 5B (S5 Table) accounted for the largest BVR in the model by including a direct effect between culture and PCR (BVR = 12.9). After controlling for this association, an additional association between culture and the clinical case definition was identified (BVR = 6.4). The LCA model controlling for both of these associations had good fit (L^2^ = 7.0; p-value = 0.99), and the probability of classification errors was 0.0%. However, 37.5% (3/8) of culture- and/or PCR-positive participants were classified as non-cases and 100% of acute and/or convalescent serology positive participants were classified as cases (S6 Table), which biased the sensitivity estimates for all diagnostic measures in the model (S5 Table).

## Discussion

Our results demonstrate that the time since cough onset should be considered when determining which laboratory test to use. The sensitivity of culture and PCR decreased from greater than 70% and 80%, respectively, during the first two weeks following cough onset to 10% and 21%, respectively after the second week following cough onset. The IgG-PT ELISA was most sensitive > 2 weeks after cough onset. In contrast, serology performed on serum collected during the first two weeks following cough onset demonstrated unacceptably low sensitivity (13%-24%). Additionally, there were two study participants with positive cultures that failed to mount a detectable antibody response between their acute and convalescent specimens, emphasizing that serology is an imperfect diagnostic tool. Despite this limitation, serology provides an additional option for patients presenting to the physician late in their illness. Similarly, data from IgG-PT ELISAs used for routine diagnosis in Massachusetts and São Paulo, Brazil have demonstrated that the inclusion of serology provides a more accurate estimate of the true burden of disease by identifying adolescent and adult pertussis infections that were missed by culture and PCR [3, 26, 27].

Traditionally, the role of a gold standard test was assigned to pertussis culture, which requires the collection of viable bacteria from the nasopharynx. However, we found that culture was only 64% sensitive in Model 1, which is similar to previously reported estimates [3, 8]. Due to the insensitivity of culture, sensitivity and specificity estimates were also calculated using CRS and LCA, which attempt to reduce gold standard bias by incorporating information from additional diagnostic measures [24]. Despite using different methodologies, CRS and LCA tended to provide similar estimates for serology and the clinical case definition within each model.

Although CRS and LCA methods reduce gold standard bias, they do not eliminate this bias completely and are subject to other limitations. Since culture and PCR are most sensitive during the first few weeks of cough [13], CRS may not have corrected the potential misclassification of participants who enrolled several weeks after cough onset. With the exception of Model 2, LCA models tended to classify participants with negative culture and PCR results, but with positive convalescent serology, as non-cases; if these participants had pertussis, this classification would bias upwards the sensitivity of PCR and culture, while biasing downwards specificity estimates for serology. This bias likely explains the apparent exaggerated estimates of culture sensitivity in Models 4 and 5A. Additionally, the low prevalence of pertussis in the study population resulted in wide confidence intervals for all of our sensitivity and specificity estimates.

While LCA is based on the assumption that all diagnostic measures are independent of one another, this assumption is rarely met in practice [28]. Failure to account for associations between diagnostic measures produces a poor fitting model that gives too much weight to those diagnostic measures [28, 29]. Although including a direct effect will improve model fit, it may also change how cases are classified by the model. As demonstrated by Model 5B, which failed to account for the association between acute and convalescent serology, over 1/3 of culture-positive participants were incorrectly classified as non-cases, which resulted in biased sensitivity and specificity estimates for all diagnostic measures in the model. In contrast, Model 5A accounted for this association and correctly classified all culture-positive participants as cases. Thus, case classification should be examined in addition to model fit statistics to minimize bias.

Our analyses were limited by missing data on diagnostic test results. The convalescent serology result was frequently missing for those who enrolled ≤ 2 weeks after cough onset since it required participants to return at a later date to provide a second blood specimen. Indeterminate PCR or serology results were excluded from these analyses. The indeterminate PCR category indicates specimens containing less than one bacterial genome, which may either indicate truly infected persons or a false-positive result [14], and the indeterminate serology category may represent either an acute, recent infection, or possibly, prior vaccination [23]. Thus, these participants’ specimens were not able to be confidently classified as either positive or negative. Additionally, we were unable to assess the effect of age, previous antibiotic usage, and pertussis vaccination status on the performance of each diagnostic test due to the low number of positive clinical specimens in the study population and missing data (i.e., LCA models did not include these variables). Not controlling for these factors in the analysis may have affected our sensitivity and specificity estimates. Additional studies should be conducted to further understand the effect of age, previous antibiotic usage, and vaccination status on the performance of pertussis diagnostic tests.

Our analyses included two serology-positive participants who were vaccinated less than six months prior to specimen collection. As both were culture- and PCR-negative, potential false positive serology results for these two participants had little impact on the model classifications or the resulting sensitivity and specificity estimates. In an earlier study to estimate the effect of Tdap vaccination on serodiagnosis of pertussis infection, antibody titers rapidly declined below the diagnostic cutoff (94 EU/mL) by 75 days post-vaccination; a six-month waiting period between vaccination and serology testing was suggested to account for potential variations in antibody response following receipt of different vaccine formulations [22].

Surprisingly, there was no difference in the proportion of participants with positive culture results between those with and without previous antibiotic use. It is impossible to determine whether this finding is related to data quality issues (i.e., participants not reporting prior antibiotic use) or another explanation. Additionally, when the latent class modeling was restricted to those without prior antibiotic use, the overall results were similar (see S7 Table). For these reasons, participants with self-reported antibiotic use in the month prior to specimen collection were included in our main latent class models. Since previous antibiotic use was expected to decrease the sensitivity of culture [9] and PCR [30], the inclusion of participants with possible recent antibiotic use in the latent class models possibly resulted in biased estimates as described earlier.

Our sensitivity and specificity findings should not be viewed as generalizable to all commercially available pertussis diagnostic tests, as PCR and serology both suffer from a lack of standardization across US laboratories. PCR practices vary by DNA extraction method, PCR targets, and diagnostic cutoff values used [11, 12, 16, 31], which contribute to differences in analytic sensitivity between assays [11, 31]. PCR assays also vary in their ability to detect other *Bordetella* species, which has resulted in the misidentification of other *Bordetella* species as *B*. *pertussis* [16, 32]. Similarly, commercial serological assays utilize various combinations of antibodies, pertussis antigens, and diagnostic cutoff values, and are not all calibrated to international reference standards [33–35]. This variability has prevented the inclusion of serology as a confirmatory laboratory test in the United States [5]. A recent analysis of commercial assays in the United States found that assays with the best performance qualities were IgG-PT assays that were calibrated to a reference standard [19]. This message appears to be reaching the commercial manufacturers, as more and more calibrated, IgG-PT assays are appearing on the market to date, suggesting that potential harmonization of serologic assays may be possible. Ideally, in the near future, potential FDA approval of these high performing assays would then allow for increased clinical utility in the United States market and improve serodiagnostic practices throughout the country.

Overall, our findings demonstrate the importance of the timing of clinical specimen collection for the diagnosis of pertussis, and the need for an accurate, standardized diagnostic test that can be used several weeks after cough onset. Consideration should be given to including IgG-PT serology as a confirmatory test for non-recently vaccinated patients with specimens collected > 2 weeks after cough onset in the CSTE case definition for pertussis to identify additional cases, especially among adolescents and adults with clinical symptoms of pertussis who seek treatment after the optimal time period for culture and PCR testing. With the recent resurgence of pertussis, it is important that we further our understanding of the epidemiology and transmission of *B*. *pertussis* in the United States, and continue evaluating current pertussis control strategies through the appropriate use of highly accurate and validated diagnostic tests.

## Supporting information

S1 Table Diagnostic measure result patterns and case classification of participants in Model 2 (n = 281). Participants in Model 2 enrolled in the study > 2 weeks after cough onset. Positive test results are indicated by (+), and negative test results are indicated by (−). Participants with missing data or indeterminate PCR or convalescent serology results were excluded from the analysis. The LCA model contains a direct effect between culture and PCR.
(PDF)

S2 Table Diagnostic measure result patterns and case classification of participants in Model 3 (n = 347). Participants in Model 3 enrolled in the study ≤ 2 weeks after cough onset. Positive test results are indicated by (+), and negative test results are indicated by (−). Participants with missing data or indeterminate PCR or convalescent serology results were excluded from the analysis.
(PDF)

S3 Table Diagnostic measure result patterns and case classification of participants in Model 4 (n = 264). Participants in Model 4 enrolled in the study ≤ 2 weeks after cough onset and returned for collection of a convalescent blood specimen. Positive test results are indicated by (+), and negative test results are indicated by (−). Participants with missing data or indeterminate PCR or convalescent serology results were excluded from the analysis.
(PDF)

S4 Table Diagnostic measure result patterns and case classification of participants in Model 5A (n = 258). Participants in Model 5A enrolled in the study ≤ 2 weeks after cough onset and had both acute and convalescent blood specimens collected. Positive test results are indicated by (+), and negative test results are indicated by (−). Participants with missing data or indeterminate PCR or convalescent serology results were excluded from the analysis. The LCA model contains a direct effect between acute and convalescent serology.
(PDF)

S5 Table Sensitivity and specificity estimates of *B*. *pertussis* diagnostic measures in participants in Model 5B (n = 258). Participants in Model 5B enrolled in the study ≤ 2 weeks after cough onset and had both acute and convalescent blood specimens collected. The LCA model contains direct effects between culture and PCR, and culture and the clinical case definition.
(PDF)

S6 Table Diagnostic measure result patterns and case classification of participants in Model 5B (n = 258). Participants in Model 5B enrolled in the study ≤ 2 weeks after cough onset and had both acute and convalescent blood specimens collected. Positive test results are indicated by (+), and negative test results are indicated by (−). Participants with missing data or indeterminate PCR or convalescent serology results were excluded from the analysis. The LCA model contains direct effects between culture and PCR, and culture and the clinical case definition.
(PDF)

S7 Table Sensitivity and specificity estimates of *B*. *pertussis* diagnostic measures in participants in Model 6 (n = 434). Participants in Model 6 had no reported antibiotic use one month prior to specimen collection, and enrolled in the study 1–29 days after cough onset. The latent class analysis (LCA) model contains a direct effect between convalescent serology and the clinical case definition.
(PDF)
