# Supplementary material for: Clinical evaluation and validation of laboratory methods for the diagnosis of Bordetella pertussis infection: Culture, polymerase chain reaction (PCR) and anti-pertussis toxin IgG serology (IgG-PT)
Source: PLoS One. 2018 Apr 13;13(4):e0195979. doi: 10.1371/journal.pone.0195979 (PMC5898745; doi:10.1371/journal.pone.0195979)
Supplement: S1 Table — Participants in Model 2 enrolled in the study > 2 weeks after cough onset. Positive test results are indicated by (+), and negative test results are indicated by (−). Participants with missing data or indeterminate PCR or convalescent serology results were excluded from the analysis. The LCA model contains a direct effect between culture and PCR. (PDF) [file pone.0195979.s001.pdf]

Culture	PCR	Convalescent serology[a]	Clinical case	N	Probability of having pertussis	Classification
−	−	−	−	106	0.0015	Non-case (n=231)
−	−	−	+	116	0.0739	
−	−	+	−	9	0.0659	
−	−	+	+	39	0.7853	Case (n=50)
−	+	+	+	6	0.9998	
+	+	−	+	2	0.9987	
+	+	+	+	3	1.0000	

[a] Convalescent sera are collected > 2 weeks after cough onset
