# Supplementary material for: Clinical evaluation and validation of laboratory methods for the diagnosis of Bordetella pertussis infection: Culture, polymerase chain reaction (PCR) and anti-pertussis toxin IgG serology (IgG-PT)
Source: PLoS One. 2018 Apr 13;13(4):e0195979. doi: 10.1371/journal.pone.0195979 (PMC5898745; doi:10.1371/journal.pone.0195979)
Supplement: S2 Table — Participants in Model 3 enrolled in the study ≤ 2 weeks after cough onset. Positive test results are indicated by (+), and negative test results are indicated by (−). Participants with missing data or indeterminate PCR or convalescent serology results were excluded from the analysis. (PDF) [file pone.0195979.s002.pdf]

Culture	PCR	Acute serology[a]	Clinical case	N	Probability of having pertussis	Classification
−	−	−	−	247	0.0011	Non-case (n=332)
−	−	−	+	72	0.0057	
−	−	+	−	11	0.0041	
−	−	+	+	2	0.0211	
−	+	−	−	1	0.9360	Case (n=15)
−	+	−	+	2	0.9870	
+	−	−	−	1	0.7879	
+	−	−	+	1	0.9506	
+	+	−	−	4	1.0000	
+	+	−	+	4	1.0000	
+	+	+	+	2	1.0000	

[a] Acute sera are collected ≤ 2 weeks after cough onset
