# Supplementary material for: Clinical evaluation and validation of laboratory methods for the diagnosis of Bordetella pertussis infection: Culture, polymerase chain reaction (PCR) and anti-pertussis toxin IgG serology (IgG-PT)
Source: PLoS One. 2018 Apr 13;13(4):e0195979. doi: 10.1371/journal.pone.0195979 (PMC5898745; doi:10.1371/journal.pone.0195979)
Supplement: S3 Table — Participants in Model 4 enrolled in the study ≤ 2 weeks after cough onset and returned for collection of a convalescent blood specimen. Positive test results are indicated by (+), and negative test results are indicated by (−). Participants with missing data or indeterminate PCR or convalescent serology results were excluded from the analysis. (PDF) [file pone.0195979.s003.pdf]

| Culture | PCR | Convalescent serology[a] | Clinical case | N | Probability of having pertussis | Classification |
|---|---|---|---|---|---|---|
| − | − | − | − | 190 | 0.0001 | Non-case (n=256) |
| − | − | − | + | 56 | 0.0006 | |
| − | − | + | − | 8 | 0.0023 | |
| − | − | + | + | 2 | 0.0199 | |
| + | − | − | + | 1 | 0.9854 | Case (n=8) |
| + | + | − | − | 1 | 1.0000 | |
| + | + | − | + | 1 | 1.0000 | |
| + | + | + | − | 1 | 1.0000 | |
| + | + | + | + | 4 | 1.0000 | |

[a] Convalescent sera are collected > 2 weeks after cough onset
