# Supplementary material for: Clinical evaluation and validation of laboratory methods for the diagnosis of Bordetella pertussis infection: Culture, polymerase chain reaction (PCR) and anti-pertussis toxin IgG serology (IgG-PT)
Source: PLoS One. 2018 Apr 13;13(4):e0195979. doi: 10.1371/journal.pone.0195979 (PMC5898745; doi:10.1371/journal.pone.0195979)
Supplement: S4 Table — Participants in Model 5A enrolled in the study ≤ 2 weeks after cough onset and had both acute and convalescent blood specimens collected. Positive test results are indicated by (+), and negative test results are indicated by (−). Participants with missing data or indeterminate PCR or convalescent serology results were excluded from the analysis. The LCA model contains a direct effect between acute and convalescent serology. (PDF) [file pone.0195979.s004.pdf]

Culture	PCR	Acute serology[a]	Convalescent serology[b]	Clinical case	N	Probability of having pertussis	Classification
−	−	−	−	−	186	0.0001	Non-case (n=250)
−	−	−	−	+	54	0.0006	
−	−	−	+	−	2	0.0064	
−	−	+	+	−	6	0.0011	
−	−	+	+	+	2	0.0098	
+	−	−	−	+	1	0.9851	Case (n=8)
+	+	−	−	−	1	1.0000	
+	+	−	−	+	1	1.0000	
+	+	−	+	−	1	1.0000	
+	+	−	+	+	2	1.0000	
+	+	+	+	+	2	1.0000	

[a] Acute sera are collected ≤ 2 weeks after cough onset

[b] Convalescent sera are collected > 2 weeks after cough onset
