# Supplementary material for: Clinical evaluation and validation of laboratory methods for the diagnosis of Bordetella pertussis infection: Culture, polymerase chain reaction (PCR) and anti-pertussis toxin IgG serology (IgG-PT)
Source: PLoS One. 2018 Apr 13;13(4):e0195979. doi: 10.1371/journal.pone.0195979 (PMC5898745; doi:10.1371/journal.pone.0195979)
Supplement: S5 Table — Participants in Model 5B enrolled in the study ≤ 2 weeks after cough onset and had both acute and convalescent blood specimens collected. The LCA model contains direct effects between culture and PCR, and culture and the clinical case definition. (PDF) [file pone.0195979.s005.pdf]

NP specimen collection timeframes	Diagnostic measures	# Positive	Latent class analysis	
			Sensitivity (95% CI)	Specificity (95% CI)
**Model 5B** ≤ 2 weeks after cough onset, n=258	**Culture**	8	32.4 (9.1- 55.8)	98.8 (97.4- 100)
	**PCR**	7	32.4 (9.1- 55.8)	99.2 (98.1- 100)
	**Acute serology**[a]	10	63.7 (39.2- 88.1)	100 (99.9- 100)
	**Convalescent serology**[b]	15	95.3 (82.2- 100)	100 (99.7- 100)
	**Clinical case**	62	39.4 (15.0- 63.8)	77.0 (71.7- 82.3)

[a] Acute sera are collected ≤ 2 weeks after cough onset

[b] Convalescent sera are collected > 2 weeks after cough onset
