# Supplementary material for: Clinical evaluation and validation of laboratory methods for the diagnosis of Bordetella pertussis infection: Culture, polymerase chain reaction (PCR) and anti-pertussis toxin IgG serology (IgG-PT)
Source: PLoS One. 2018 Apr 13;13(4):e0195979. doi: 10.1371/journal.pone.0195979 (PMC5898745; doi:10.1371/journal.pone.0195979)
Supplement: S6 Table — Participants in Model 5B enrolled in the study ≤ 2 weeks after cough onset and had both acute and convalescent blood specimens collected. Positive test results are indicated by (+), and negative test results are indicated by (−). Participants with missing data or indeterminate PCR or convalescent serology results were excluded from the analysis. The LCA model contains direct effects between culture and PCR, and culture and the clinical case definition. (PDF) [file pone.0195979.s006.pdf]

Culture	PCR	Acute serology[a]	Convalescent serology[b]	Clinical case	N	Probability of having pertussis	Classification
−	−	−	−	−	186	0.0008	Non-case (n=243)
−	−	−	−	+	54	0.0008	
+	−	−	−	+	1	0.0004	
+	+	−	−	−	1	0.0430	
+	+	−	−	+	1	0.0431	
−	−	−	+	−	2	0.9836	Case (n=15)
−	−	+	+	−	6	1.0000	
−	−	+	+	+	2	1.0000	
+	+	−	+	−	1	0.9997	
+	+	−	+	+	2	0.9997	
+	+	+	+	+	2	1.0000	

[a] Acute sera are collected ≤ 2 weeks after cough onset

[b] Convalescent sera are collected > 2 weeks after cough onset
