# Supplementary material for: Clinical evaluation and validation of laboratory methods for the diagnosis of Bordetella pertussis infection: Culture, polymerase chain reaction (PCR) and anti-pertussis toxin IgG serology (IgG-PT)
Source: PLoS One. 2018 Apr 13;13(4):e0195979. doi: 10.1371/journal.pone.0195979 (PMC5898745; doi:10.1371/journal.pone.0195979)
Supplement: S7 Table — Participants in Model 6 had no reported antibiotic use one month prior to specimen collection, and enrolled in the study 1–29 days after cough onset. The latent class analysis (LCA) model contains a direct effect between convalescent serology and the clinical case definition. (PDF) [file pone.0195979.s007.pdf]

NP specimen collection timeframes	Diagnostic measures	# Positive	Culture as the "gold standard"		Composite reference standard		Latent class analysis	
			**Sensitivity (95% CI)**	**Specificity (95% CI)**	**Sensitivity (95% CI)**	**Specificity (95% CI)**	**Sensitivity (95% CI)**	**Specificity (95% CI)**
**Model 6** 1-29 days after cough onset, n=434	**Culture**	10	N/A	N/A	N/A	N/A	65.5 (39.6- 91.3)	99.9 (99.2- 100)
	**PCR**	13	90.0 (71.4- 100)	99.1 (98.1- 100)	N/A	N/A	88.4 (55.3- 100)	100 (99.9- 100)
	**Convalescent serology**[a]	39	70.0 (41.6- 98.4)	92.5 (89.9- 95.0)	78.6 (57.1- 100)	93.3 (91.0- 95.7)	78.2 (51.6- 100)	93.4 (91.0- 95.9)
	**Clinical case**	161	90.0 (71.4- 100)	64.2 (59.6- 68.7)	92.9 (79.4- 100)	64.8 (60.2- 69.3)	90.7 (75.5- 100)	64.8 (60.2- 69.4)

Abbreviations: NP, nasopharyngeal; 95% CI, 95% Confidence Interval; N/A, not applicable

[a] Convalescent sera are collected > 2 weeks after cough onset. Participants with NP specimens collected ≤ 2 weeks after cough onset returned 2-4 weeks later to provide the convalescent blood specimen
